# Feasibility and effectiveness of a manualized group psychotherapy program for autistic adults: a naturalistic clinical study

**DOI:** 10.3389/fpsyt.2026.1863942

**Published:** 2026-08-05

**Authors:** Tina Schweizer, Thomas Fangmeier, Andrea Lichtblau, Julia Peters, Manon Mannherz, Monica Biscaldi-Schäfer, Dieter Ebert, Andreas Riedel, Ludger Tebartz van Elst

**Affiliations:** 1 Department of Psychiatry and Psychotherapy, Medical Center - University of Freiburg, Faculty of Medicine, University of Freiburg, Freiburg, Germany; 2 Department of Child and Adolescent Psychiatry, Psychotherapy, and Psychosomatics, Medical Center, Faculty of Medicine, University of Freiburg, Freiburg, Germany

**Keywords:** autism spectrum disorder, autistic adults, psychosocial intervention, group psychotherapy, social functioning, depression, social skills, stress management

## Abstract

**Background:**

Psychosocial interventions for autistic adults remain limited. This study evaluated the implementation feasibility and potential clinical effectiveness of a manualized group psychotherapy program delivered under routine care conditions.

**Methods:**

Data from 115 autistic adults who completed the program in routine outpatient care were analyzed. Outcomes included pre- to post-intervention changes in social responsiveness as primary outcome, as well as self-worth, depressive symptoms, and quality of life as secondary outcomes.

**Results:**

Overall social responsiveness improved significantly from pre- to post-intervention. Significant post-intervention improvements were further detected for self-worth and depressive symptoms, while quality of life showed no significant change.

**Discussion:**

The findings indicate that the manualized group psychotherapy program seems to be feasible in routine clinical care and was associated with improvements in social functioning, self-worth, and depressive symptoms, suggesting preliminary clinical effectiveness. However, randomized controlled trials are warranted to further establish efficacy.

## 1 Introduction

For more than a decade, there has been growing recognition that autism spectrum disorder (ASD) is not only relevant in child and adolescent psychiatry but also represents an important clinical condition in adult psychiatry and psychotherapy (1–4). ASD is a neurodevelopmental condition characterized by persistent differences in social interaction and communication, as well as restricted and repetitive patterns of behavior and interests. Epidemiological studies estimate the prevalence of ASD to range between 1-2% in the general population (2, 5, 6). Males are more frequently diagnosed than females, with reported male-to-female ratios ranging from 3.4:1 to 4.3:1 (6, 7).

One explanation for this discrepancy is that, in autistic adults without intellectual disability, females may present with an ASD phenotype that differs from that typically observed in males, potentially leading to under-recognition in clinical settings (8, 9). Camouflaging strategies, which are particularly common in autistic females, may further mask autistic traits and contribute to delayed or missed diagnoses (10, 11). Consequently, autistic women in particular may be diagnosed less frequently or at a later age in clinical practice. In addition, misdiagnosis is relatively common among autistic adults, who are sometimes assigned alternative psychiatric diagnoses (12–14).

Furthermore, psychiatric comorbidity in autistic adults without intellectual disability is consistently reported to be high, particularly with regard to anxiety and depressive disorders (15–17).

While some psychiatric conditions may develop independently of ASD, ASD-related characteristics may in many cases contribute to the development or maintenance of psychiatric comorbidity. Characteristic autistic behaviors - such as reduced eye contact or avoidance of small talk - may lead to misunderstandings or irritation among non-autistic individuals (18). Many autistic adults also experience difficulties in recognizing and interpreting others’ emotions or in expressing their own, and their often concrete or literal cognitive processing styles may further complicate social communication and lead to interpersonal conflicts (19–22). Persistent challenges in social interaction and communication may also partly explain lower levels of occupational participation and social integration compared with the general population, even among those with average or above-average intellectual abilities, which in turn may contribute to depressive symptoms (23, 24). In addition, heightened sensitivity to sensory and social stimuli can result in increased stress levels in everyday social contexts (25–27). Moreover, this population often experiences distinct challenges related to affective and sexual functioning, frequently including difficulties with implicit social expectations, initiating dating interactions, interpreting interpersonal cues, and managing anxiety related to intimacy, which may additionally contribute to psychosocial stress and mental health burden (28, 29).

Consequently, many autistic adults experience persistent challenges in daily functioning, mental health, and social participation, highlighting the clinical relevance of developing targeted interventions for this population.

In this context, ASD may be conceptualized as a core vulnerability structure underlying the development of psychiatric comorbidities. Similarly, in individuals with subsyndromal traits, a broader autism phenotype may contribute to increased vulnerability to psychiatric disorders (4).

Taken together, these findings underscore the substantial need for effective interventions specifically tailored to autistic adults without intellectual disability.

Currently, there is no licensed pharmacological treatment specifically targeting the core social and communicative difficulties experienced by autistic adults. Oxytocin, one of the most extensively studied pharmacological candidates, has yielded mixed findings, with some studies suggesting that chronic administration may even impair social bonding processes (30–32).

Regarding psychosocial approaches, only a limited number of programs specifically addressing social functioning and stress management have been developed and evaluated for autistic adults without intellectual disability (33–38). Empirical studies further indicate a substantial demand for interventions tailored to the needs of autistic adults (39). Among the most established interventions in this area are the PEERS and ACCESS programs, both of which were developed to support autistic adolescents and young adults during the transition to adulthood and employ highly structured, autism-adapted group formats incorporating cognitive-behavioral principles (35, 40–42). The PEERS program represents the most extensively studied intervention to date, whereas the ACCESS program constitutes another promising structured group intervention for autistic young adults. The PEERS program primarily focuses on the acquisition and rehearsal of concrete social skills required for initiating and maintaining peer relationships, including conversational abilities, friendship development, conflict management, and coping with teasing or bullying. In contrast, the ACCESS program places greater emphasis on coping strategies, adaptive functioning, stress management, and self-determination during the transition to adulthood.

Despite these advantages, most existing interventions are based on relatively small samples, and robust evidence regarding their clinical efficacy and effectiveness remains limited, highlighting the need for further research evaluating such interventions (e.g. 43). In particular, manualized group psychotherapy is recommended as the first-line intervention for autistic adults without intellectual disability in the German treatment guideline (44).

To address this gap, the Freiburg Asperger-Specific Therapy Program for Adults (FASTER) was developed as a manualized group psychotherapy program integrating psychoeducation, stress management, and structured training of social interaction and communication skills. The primary aim of the FASTER program is to support autistic adults in managing challenges related to social communication and interaction, while strengthening stress management and coping strategies. Similar to the PEERS and ACCESS program, FASTER applies a structured and autism-adapted group framework; however, its therapeutic focus lies more strongly on the interaction between social functioning, stress regulation, and self-awareness across a broader age range of autistic adults without intellectual disability within routine clinical care settings.

While first studies suggested feasibility and potential effectiveness in small patient cohorts (33, 34), systematic evaluation in larger samples is still lacking.

Thus, this study aimed to systematically evaluate the implementation feasibility and potential clinical effectiveness of the manualized FASTER group psychotherapy program in a larger cohort under routine care conditions.

## 2 Materials and methods

### 2.1 Study design

This study employed a naturalistic, single-arm pre–post intervention design to evaluate the implementation feasibility and potential clinical effectiveness of a manualized group psychotherapy program for autistic adults without intellectual disability who have good language skills and lower support needs. Feasibility was evaluated in terms of implementation under routine clinical care conditions and assessed through treatment attendance, completion rates, and dropout across the intervention period. Hypotheses, outcome measures, assessment time points, and analyses addressing the predefined research questions were planned *a priori*. This design allows examination of changes between pre- (T0) and post-intervention (T1) assessments under real-world clinical conditions, reflecting routine care settings and providing ecologically valid insights into the practical implementation of the therapy.

### 2.2 Participants

At the Freiburg Centre for Autism Spectrum Disorders (German: Universitäres Zentrum Autismus Spektrum, UZAS; https://www.uniklinik-freiburg.de/psych/klinische-schwerpunkte/asperger-autismus.html) FASTER group psychotherapy has been offered to outpatients since 2008. Patients were eligible if they were aged 18–65 years, with no intellectual disability (IQ > 70), good language skills, lower support needs, and an established diagnosis of autism spectrum disorder (ASD; ICD-10 F84.0, F84.1, or F84.5). Diagnostic procedures comprised comprehensive clinical assessment, including developmental history, detailed clinical evaluation, and the use of standardized diagnostic instruments (e.g., AQ, EQ, ADOS-2), as well as multidisciplinary clinical consensus. Where clinically indicated, additional neuropsychological assessments were conducted. Autism diagnoses were established at the Freiburg Centre for Autism Spectrum Disorders in accordance with the German AWMF S3 guidelines in effect at the time (for a more detailed description, see 3). Diagnoses obtained from other centers were systematically reviewed by the UZAS diagnostic team prior to inclusion. For this study, data from seventeen therapy groups were included.

Exclusion criteria comprised subsyndromal autism symptoms (“broader autism phenotype”), severe behavioral disturbances interfering with group participation, florid psychotic or bipolar disorder, current substance use disorder, severe depressive symptoms or suicidality, and any neurological or medical condition likely to interfere with participation in group therapy.

To ensure meaningful participation, patients were required to attend at least 25% of therapy sessions. On average, groups comprised 31 sessions, corresponding to a minimum of eight sessions per participant. Participation in at least 25% of sessions was defined as the minimum criterion for treatment exposure. This threshold was selected pragmatically to ensure at least partial exposure to the intervention while retaining sufficient sample size under naturalistic routine care conditions and was not intended to reflect an adequate therapeutic dose or treatment completion criterion.

**Figure 1:**
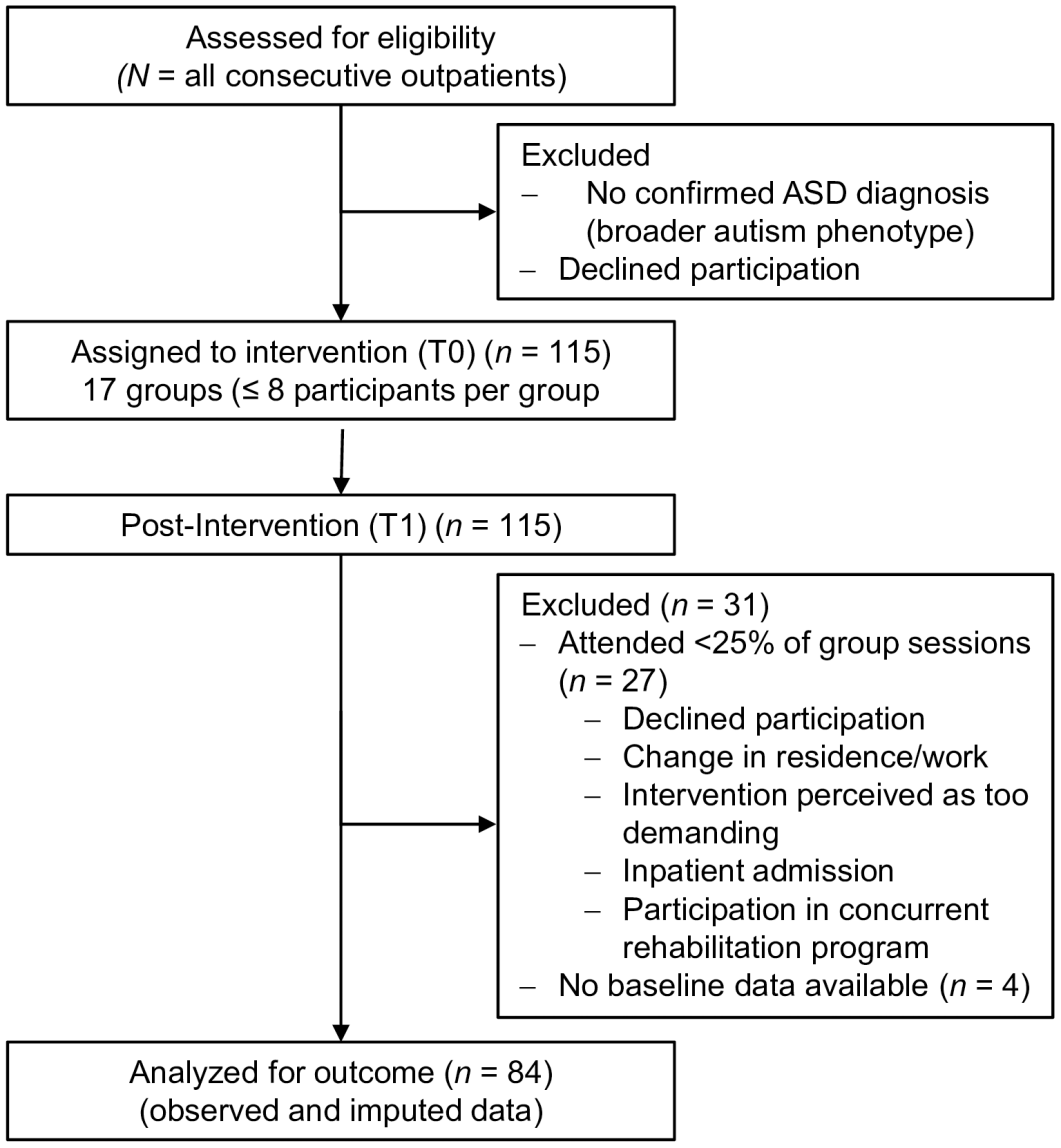
Flowchart of participant inclusion in the FASTER group psychotherapy program. A total of 17 groups conducted between 2008 and 2018 were included. Of the 115 participants initially enrolled, 84 were included in the final analysis based on the following criteria: a confirmed ASD diagnosis, attendance of at least 25% of psychotherapy sessions, and availability of baseline questionnaires.

The final sample included 84 of the initial 115 participants (see flowchart in **Figure 1**), of whom 28.6% were female (*n* = 24). Mean age was 34 years (*M* = 34.1; range: 18–58). ASD diagnoses according to ICD-10 comprised atypical autism (*n* = 12, 14.3%), early childhood autism (*n* = 6, 7.1%), and Asperger syndrome (*n* = 66, 78.6%). A substantial proportion of participants presented with comorbid psychiatric diagnoses, with multiple diagnoses per participant possible. The most common were affective disorders (*n* = 35, 41.7%), followed by obsessive–compulsive disorders (*n* = 5, 6%), and substance use disorders, anxiety disorders, and post-traumatic stress disorder (each; *n* = 3, 3.6%).

### 2.3 Intervention

The FASTER program is a manualized, autism-specific group psychotherapy program developed for autistic adults without intellectual disability, good language skills, and lower support needs (3, 33, 34). It was designed to address the needs of autistic adults who frequently present to adult psychiatric services primarily because of co-occurring conditions such as depression and anxiety (45, 46). The intervention aims to support autistic adults in managing challenges related to social communication and interaction, improving self-regulation, stress management abilities, emotion recognition and interpersonal awareness, and facilitating the transfer of learned strategies into everyday functioning.

FASTER is primarily delivered in a group format, based on the rationale that social communication difficulties are best addressed in ecologically relevant interpersonal contexts. The group setting provides opportunities to observe, practice, and reflect on social interactions in a structured and supportive environment while facilitating peer exchange and social learning.

The intervention integrates selected elements informed by cognitive behavioral therapy (CBT) and dialectical behavior therapy (DBT) approaches. Elements informed by CBT include psychoeducation, and structured training of social communication and interaction skills, based on established social skills training approaches. To address core challenges in social communication and interaction, these elements are delivered through structured exercises, role-play, and individualized feedback procedures. Exercises gradually increase in complexity and involve progressively reduced external structure, while emphasizing repetition and gradual transfer to more naturalistic social situations to promote behavioral flexibility and skills generalization. Role-plays are combined with structured individualized feedback from therapists and peers to support self-awareness, perspective-taking, recognition of social cues, attention to interpersonal dynamics, and constructive feedback. DBT-informed elements include mindfulness-based techniques and strategies for recognizing and managing stress and emotional overload. These techniques were selected to address challenges related to recognizing internal states and coping with stress and were adapted to support the recognition of personal limits, facilitate flexible attentional focus, and improve the identification and management of stress and overload in in socially and sensory demanding situations.

The intervention is delivered in groups of 6–8 participants and comprises approximately 31 weekly 120-minute sessions over eight months, organized into three sequential treatment modules. Sessions follow a fixed weekly schedule and a highly structured, consistent format to enhance predictability and reduce uncertainty, which is considered particularly relevant for autistic adults. The intervention followed a manualized therapeutic structure and was delivered by therapists who had received prior training in the FASTER treatment approach. The standardized session structure includes mood check-ins, introduction and practice of session topics, and a closing feedback round. Deviations from the schedule are minimized and communicated in advance when necessary.

The basic module focuses on psychoeducation on ASS, group rules and goals, and autism-related strengths and challenges, using a strengths-based and acceptance-oriented approach to support self-understanding. The second module targets foundational verbal and non-verbal communication skills, emotion recognition, and stress awareness through behavioral and situational analyses. In addition, mindfulness-based techniques are introduced to support emotional self-awareness, recognition of personal limits, and stress management. The final module focuses on increasingly complex communication and interpersonal situations and emphasizes behavioral rehearsal through structured role-play exercises targeting everyday social interactions. Participants practice every day social situations, including initiating and ending conversations or telephone calls, small talk, interpersonal relationships, and coping with social overload.

Autism-specific adaptations principles included a highly structured and predictable treatment format, a strengths-based and acceptance-oriented psychoeducational approach, gradual progression from structured to increasingly flexible interpersonal exercises, repeated practice to support skills generalization.

The initial program underwent iterative refinements based on clinical experience and patient feedback before its formal implementation. Detailed descriptions of the FASTER intervention have been published previously (33, 34).

### 2.4 Outcome measures

#### 2.4.1 Primary outcome measure

Given that ASD is fundamentally characterized by persistent challenges in reciprocal social interaction and communication, the Social Responsiveness Scale (SRS; informant-report) was selected as the primary outcome measure (47). An informant report was used to capture observable aspects of social behavior and interpersonal functioning, which are central to autism and may be less accurately reflected in self-report. This approach was intended to provide an ecologically valid assessment of changes in everyday social interactions. The SRS has been widely used in studies targeting social responsiveness in ASD and has demonstrated good psychometric properties and has been validated as a dimensional measure of autism-related social impairment across a wide age range, including adults (48–53). The German version has demonstrated good reliability and validity in previous studies (Cronbach’s α = .89; 54). Analyses focused primarily on the total score as well as the five subscale scores - social awareness, social cognition, social communication, social motivation, and autistic mannerisms.

Furthermore, solely as an ancillary, exploratory measure, an adapted version of the SRS was used (SRS mod) to assess perceived changes relative to baseline, including the original 65 items with a modified response scale. Participants rated each item on an adapted five-point Likert scale: *very much deteriorated* (-2), *somewhat deteriorated* (-1), *remained the same* (0), *somewhat improved* (+1), and *very much improved* (+2).

#### 2.4.2 Secondary outcome measures

Autistic adults exhibit significantly elevated rates of psychiatric comorbidities, particularly depressive disorders, often fueled by chronic masking, stress, and social exclusion. These psychological burdens inherently compromise functional adaptation and subjective well-being. Consequently, cross-domain secondary outcome measures were tracked to assess broader therapeutic generalization beyond core social responsiveness. Regarding self-worth, general psychological resilience and self-appraisal were captured via the Multidimensional Self-Worth Scale (MSWS), which evaluates cognitive-affective facets of personal value. Depressive symptoms were quantified using the Beck Depression Inventory (BDI), a self-report questionnaire evaluating the severity of current depressive symptoms. Finally, overall subjective well-being was measured with the World Health Organization Quality of Life (WHOQOL) questionnaire, assessing physical, psychological, and social-environmental domains of quality of life.

All questionnaires related to the primary and secondary outcomes, were established standardized instruments and administered at both baseline and post-intervention to assess changes over time associated with the intervention.

### 2.5 Statistical analysis

Missing data were handled using multiple imputation with 20 generated datasets under the assumption of missing at random (MAR), in line with recommended practice (55, 56). Given the relatively high attrition rate and missingness across outcome measures, multiple imputation was applied to reduce potential bias associated with incomplete observations and maximize retention of available information. All variables with two measurement time points and available baseline data were entered into the imputation model. Imputed values were checked and restricted to plausible minimum and maximum values of the respective questionnaires.

Longitudinal changes in primary and secondary outcome measures were analyzed using linear mixed-effects models with maximum likelihood estimation in Stata 17. Mixed-effects models were conducted on the imputed datasets to estimate pre–post changes while accounting for repeated observations within participants. The dependent variables were the total scores of the SRS, MSWS, BDI, and WHOQOL. Time (T0 *vs*. T1) was included as a fixed effect, with random intercepts specified for participants. Estimates across imputed datasets were combined according to Rubin’s rules. The main outcome measure (SRS total score) was evaluated without adjustment for multiple comparisons. Bonferroni correction was applied to all secondary outcome analyses to account for multiple testing.

Sensitivity analyses using non-imputed observed data were additionally conducted for the primary and secondary outcome measures to examine the robustness of findings across different approaches to handling missing data.

Wilcoxon signed-rank tests were conducted to examine perceived post-intervention change scores (T1 relative to baseline), testing whether scores differed significantly from zero for the adapted Social Responsiveness Scale (SRS mod) as an ancillary, exploratory measure. Bonferroni correction was applied to account for multiple comparisons across SRS mod subscales.

All tests were two-tailed with a significance level of α = .05.

### 2.6 Ethics

The study was approved by the Ethics Committee of the University of Freiburg (approval number: 26-1045-S1-retro) and conducted in accordance with institutional ethical standards and the principles of the Declaration of Helsinki. Hypotheses, outcome measures, assessment time points, and statistical analyses addressing the predefined research questions were specified *a priori*. The study was based on secondary analysis of anonymized clinical routine data. The requirement for written informed consent was waived by the ethics committee due to the retrospective analysis of fully anonymized clinical routine data. All participants had provided broad consent at admission, permitting the use of their clinical data for research purposes, in accordance with local legislation and institutional requirements.

## 3 Results

Improvements in social responsiveness were observed in the overall SRS score (*F*_(1,65.6)_ = 4.68, *p* = .034, *dz* = 0.58). No SRS subscale remained statistically significant after correction for multiple comparisons. The observed improvement in overall social responsiveness corresponded to a moderate effect size. Detailed results of the SRS analyses are presented in **Table 1**.

**Table 1:** Results of social responsiveness assessed by SRS (overall sum and subscales).

SRS	Baseline (T0)			Post-intervention (T1)		Mixed model						
	*n*	*M*	(SE)	*M*	(SE)	*F*	*df*	*p*	*t*	*dz*	CI 95% (*t*-value)	
Total	48	94.31	(4.366)	81.51	(5.571)	4.68	1, 65.6	.034*	-2.16	0.58	-24.62	-0.99
SCons	48	10.06	(0.581)	9.06	(0.757)	1.57	1, 71.6	.214	-1.25	0.33	-2.60	0.59
SCog	48	16.83	(0.948)	14.88	(1.342)	2.33	1, 53.7	.133	-1.53	0.50	-4.52	0.61
SCom	47	31.17	(1.638)	25.86	(2.158)	5.43	1, 47.7	.024	-2.33	0.68	-9.90	-0.73
SMot	46	20.02	(0.924)	18.25	(1.988)	0.69	1, 43.1	.412	-0.83	0.23	-6.08	2.54
AM	48	16.75	(0.844)	15.41	(1.492)	0.64	1, 59.0	.427	-0.80	0.18	-4.70	2.01

SRS, Social Responsiveness Scale, informant-report.

Total, total sum score; SCons, Social Consciousness; SCog, Social Cognition; SCom, Social Communication; SMot, Social Motivation; AM, Autistic Mannerism

*Note*. Mixed model included time (baseline, post-intervention) as a fixed effect and participant as a random effect. Results are based on 20 multiply imputed datasets (missing at random assumption). The SRS total score was evaluated as the primary outcome measure without adjustment for multiple comparisons. P-values for SRS subscale analyses were adjusted using Bonferroni correction. *F, df, p*, mixed model statistics; *t*, CI = *t*-statistic and 95% confidence interval; *dz*, standardized within-subject effect size for pre–post change.

*p < .05.

In addition, to more explicitly capture participants’ perceived changes in social reactivity from baseline to post-intervention, the SRS mod was used as an ancillary, exploratory measure. For further information about the modified scale, see Section 2.4. The analysis revealed significant improvements in the overall score (Wilcoxon signed-rank test, *Z* = 4.53, *p* <.001) as well as in all subscales (see **Table 2**).

**Table 2:** Results of social responsiveness assessed by SRS mod (overall sum and subscales).

SRS mod	Post-intervention (T1) relative to baseline					Wilcoxon signed rank test		Sign (*n*)			
	*n*	*M*	(SE)	CI 95%		*z*	*p*	positive	negative	zero	
Total	38	18.34	(3.765)	10.71	25.97	4.53	<.001***	34	4		0
SCons	38	1.95	(0.507)	0.92	2.98	4.44	<.001***	28	2		8
SCog	38	3.08	(0.724)	1.61	4.55	4.13	<.001***	28	4		6
SCom	38	6.39	(1.245)	3.87	8.92	4.72	<.001***	32	2		4
SMot	38	4.00	(0.816)	2.35	5.65	4.07	<.001***	28	6		4
AM	37	3.00	(0.752)	1.47	4.53	4.22	<.001***	27	3		7

SRS mod, Social Responsiveness Scale, adapted answer scale, informant-report.

Total, total sum score; SCons, Social Consciousness; SCog, Social Cognition; SCom, Social Communication; SMot, Social Motivation; AM, Autistic Mannerism

*Note*. Outcomes were assessed at post-intervention relative to baseline. The number of positive, negative, and zero differences is reported. Wilcoxon signed-rank tests were conducted to evaluate deviations from zero. P-values were adjusted using Bonferroni correction for multiple comparisons across the subscales.

***p < .001.

The overall self-worth score, assessed with the MSWS, increased significantly from pre- to post-intervention (*F*_(1, 200.7)_ = 6.68, *p* = .010; *dz* = 0.40), corresponding to a small effect size. In addition, three of the four subscales showed significant improvements: emotional self-worth (*F*_(1, 228.0)_ = 9.69, *p* = .048), self-worth related to safety in contact (*F*_(1, 253.1)_ = 5.61, *p* = .019), and performance-related self-worth (*F*_(1, 570.2)_ = 4.11, *p* = .043). The second-order subscale “general self-worth” also increased significantly (*F*_(1, 617.4)_ = 9.69, *p* = .002). Detailed results are presented in **Table 3**.

**Table 3:** Results of self-worth assessed by MSWS (overall sum and subscales).

MSWS	Baseline (T0)			Post-intervention (T1)		Mixed model						
	*n*	*M*	(SE)	*M*	(SE)	*F*	*df*	*p*	*t*	*dz*	CI 95% (*t*-value)	
Total	81	112.40	(4.055)	121.51	(4.164)	6.68	1, 200.7	.010*	2.59	0.40	2.16	16.06
ASW	81	76.86	(3.039)	83.89	(2.986)	9.69	1, 617.4	.002**	3.11	0.43	2.59	11.45
KSW	81	35.53	(1.313)	36.44	(1.459)	0.65	1, 258.3	.420	0.81	0.12	-1.31	3.13
ESWS	81	26.04	(1.207)	28.27	(1.366)	3.94	1, 228.0	.048*	1.98	0.30	0.02	4.45
SWKO	81	14.17	(0.748)	15.84	(0.834)	5.61	1, 253.1	.019*	2.37	0.40	0.28	3.05
SWKR	81	17.95	(0.964)	19.31	(0.936)	3.09	1, 325.1	.080	1.76	0.25	-0.16	2.89
LSWS	81	18.70	(0.745)	20.02	(0.775)	4.11	1, 570.2	.043*	2.03	0.28	0.04	2.59
SWPA	81	19.47	(0.765)	19.76	(0.803)	0.21	1, 309.0	.648	0.46	0.07	-0.96	1.55
SWSP	81	16.06	(0.737)	16.61	(0.848)	0.68	1, 277.2	.412	0.82	0.12	-0.76	1.85

MSWS, Multidimensional Self-worth Scales.

*Note*. Subscales 2^nd^ order: AWS, general self-worth (composed of ESWS, SWKO, SWKR, and LSWS) and KSW, body-related self-worth (composed of SWPA and SWSP). Subscales: ESWS, emotional self-worth; SWKO, self-worth - safety in contact; SWKR, self-worth - dealing with criticism; LSWS, performance-related self-worth; SWPA, physical attractiveness; SWSP =sportiness. Mixed model included time (baseline, post-intervention) as a fixed effect and participant as a random effect. Results are based on 20 multiply imputed datasets (missing at random assumption). P-values were adjusted using Bonferroni correction for multiple comparisons across secondary outcomes and, within the MSWS, across the two higher-order subscales. *F, df, p*, mixed model statistics; *t*, CI = *t*-statistic and 95% confidence interval; *dz*, standardized within-subject effect size for pre–post change.

*p < .05; **p < .01.

Depressive symptoms measured with the BDI also improved over time (*F*_(1,109.7)_ = 8.77, *p* = .004; *dz* = 0.49; see **Table 4**), corresponding to a small effect size, corresponding to a small effect size (near-moderate magnitude).

**Table 4:** Result of depressive symptoms assessed by BDI (overall sum).

BDI	Baseline (T0)			Post-intervention (T1)		Mixed model						
	*n*	*M*	(SE)	*M*	(SE)	*F*	*df*	*p*	*t*	*dz*	CI 95% (*t*-value)	
Total	81	18.30	(1.459)	14.62	(1.559)	8.77	1, 109.7	.004**	-2.96	0.49	-6.14	-1.22

BDI, Beck Depression Inventory (BDI and BDI II), overall sum.

*Note*. Mixed model included time (baseline, post-intervention) as a fixed effect and participant as a random effect. Results are based on 20 multiply imputed datasets (missing at random assumption). P-values were adjusted using Bonferroni correction for multiple comparisons across secondary outcomes. *F, df, p*, mixed model statistics; *t*, CI = *t*-statistic and 95% confidence interval; *dz*, standardized within-subject effect size for pre–post change.

**p < .01.

For quality-of-life measures assessed with the WHOQOL-BREF, no significant pre–post changes were observed for the total score (*F*_(1, 191.7)_ = 1.38, *p* = .242) or the subscales physical health (*F*_(1, 334.5)_ = 0.46, *p* = .460), psychological health (*F*_(1, 245.3)_ = 1.44, *p* = .231), social relations (*F*_(1, 350.9)_ = 0.58, *p* = .447), and environment (*F*_(1, 196.7)_ = 0.08, *p* = .781). Detailed results are presented in **Table 5**.

**Table 5:** Results of quality of life assessed by WHOQOL (overall sum and subscales).

WHOQOL-BREF	Baseline (T0)			Post-intervention (T1)		Mixed model						
	*n*	*M*	(SE)	*M*	(SE)	*F*	*df*	*p*	*t*	*dz*	CI 95% (*t*-value)	
Total	79	80.13	(1.645)	81.56	(1.812)	1.38	1, 191.7	.242	1.17	0.19	-0.98	3.85
Physical Health	80	19.63	(0.408)	19.91	(0.415)	0.46	1, 334.5	.460	0.68	0.10	-0.55	1.12
Psychological Health	79	17.54	(0.433)	18.02	(0.446)	1.44	1, 245.3	.231	1.20	0.18	-0.30	1.24
Social Relations	79	8.38	(0.309)	8.59	(0.336)	0.58	1, 350.9	.447	0.76	0.11	-0.33	0.74
Environment	80	28.74	(0.673)	28.87	(0.807)	0.08	1, 196.7	.781	0.28	0.05	-0.78	1.04

WHOQOL-BREF, World Health Organization Quality of Life – BREF (short form).

*Note.* Mixed model included time (baseline, post-intervention) as a fixed effect and participant as a random effect. Results are based on 20 multiply imputed datasets (missing at random assumption). P-values were adjusted using Bonferroni correction for multiple comparisons across secondary outcomes. *F*, *df*, *p*, mixed model statistics; *t*, CI = *t*-statistic and 95% confidence interval; *dz*, standardized within-subject effect size for pre–post change.

Across measures, the number of available observations at baseline (T0) and post-intervention (T1) varied. For the SRS (other-report), data were available for *n* = 48 participants at both time points, with missing T1 values imputed for 32 cases. For the MSWS, complete data were available for *n* = 81 participants, with 25 missing T1 values imputed. For the BDI, complete data were available for *n* = 81 participants, with 22 missing T1 values imputed. For the WHOQOL-BREF, complete data were available for *n* = 79 participants, with 22 missing T1 values imputed. For the SRS difference score (other-report), data were available for *n* = 38 participants (assessed at T1 only). Missing data were handled using multiple imputation with 20 generated datasets in line with recommended practice (55, 56). All variables included two measurement points and available baseline data were entered into the imputation model.

Exploratory baseline comparisons by sex revealed no significant differences in age or primary outcome severity. Likewise, exploratory comparisons across treatment participation levels revealed no significant differences in baseline age, sex, or primary outcome severity. However, these findings should be interpreted cautiously given the limited sample size and exploratory nature of the analyses.

Sensitivity analyses based on non-imputed observed data yielded highly comparable findings to the multiply imputed analyses (MI mixed model *p*/observed-data mixed model *p*: SRS total = .034/.011, BDI = .004/<.001; MSWS total = .010/.011; WHOQOL total = .242/.185) supporting the robustness of results across different approaches to handling missing data.

Regarding implementation feasibility, approximately 32% of participants attended fewer than 25% of sessions and did not meet the predefined minimum attendance criterion for inclusion in outcome analyses.

## 4 Discussion

This open, naturalistic, single-center study evaluated the implementation feasibility and potential clinical effectiveness of the manualized FASTER group psychotherapy program for autistic adults without intellectual disability. The findings indicate that the implementation of the program may be feasible in routine clinical care conditions and was associated with significant improvements in social responsiveness, self-worth, and depressive symptoms, thereby providing preliminary evidence for clinical effectiveness.

Consistent with this, exploratory analysis revealed that participants reported perceived improvements in social responsiveness from baseline to post-intervention, suggesting that the program was also subjectively helpful.

### 4.1 Social responsiveness

The observed improvements in social responsiveness are broadly consistent with previous evidence suggesting that psychosocial interventions for autistic adults may lead to gains in specific domains of social functioning (35, 40, 41, 57, 58). Although the existing literature is limited by small, heterogeneous samples and inconsistent outcomes (e.g. 43), prior group-based interventions generally combine psychoeducation, structured behavioral rehearsal, and cognitive-behavioral strategies, with repeated practice and feedback to support skill acquisition and generalization. This framework aligns with the conceptual approach of the FASTER program and is consistent with recommendations that effective behavioral and developmental interventions for ASD explicitly target social and communication difficulties (59). It also reflects recommendations for manualized group psychotherapy as the first-line intervention for autistic adults without intellectual disability (44). Moreover, the FASTER program is tailored to the specific needs of autistic adults, designed to create conditions that may facilitate the acquisition of new knowledge and skills in this population.

At the same time, previous research suggests that improvements in social functioning may not be exclusively attributable to highly structured skills training. Comparable gains in social cognition, social responsiveness, and functional outcomes have been observed in less structured social interaction groups (42), highlighting the potential contribution of shared social experiences and interaction with other group members, and contextualized learning as active components of change. Accordingly, the improvements observed in this study may reflect both the structured components of the intervention and the therapeutic effects of the group format itself.

The group format may be particularly relevant for autistic adults, offering opportunities to explore and practice social interactions in a structured and supportive environment while receiving feedback from both therapists and peers. Participants’ personally challenging situations could be simulated within a controlled setting, which may facilitate the generalization of acquired knowledge and skills to everyday life - a critical factor for achieving meaningful and sustained treatment effects, given that generalization to real-world contexts remains a key challenge in autism interventions.

Furthermore, improvements in social functioning may also contribute to changes in other outcome domains by fostering more positive social experiences and reinforcing self-concept.

From a clinical perspective, these findings may suggest the potential value of structured, group-based psychosocial interventions in routine care, not only for enhancing social competence but also for supporting broader psychological well-being in autistic adults.

### 4.2 Self-worth, depressive symptoms, and quality of life

Notable improvements in self-worth and a significant reduction in depressive symptoms were observed, whereas quality of life did not change significantly. This pattern is consistent with previous research suggesting that psychosocial interventions for autistic adults may effectively target proximal psychological outcomes, such as mood and self-concept, whereas effects on broader, multidimensional constructs like quality of life are often limited or inconsistent (e.g. 60). Beyond individual psychological functioning, quality of life is influenced by relatively stable environmental and social factors - such as living conditions, employment, and social participation - which are less likely to change over the course of brief interventions (61, 62).

In contrast, constructs like self-worth and depressive symptoms may be more directly influenced by structured psychosocial interventions, particularly those incorporating psychoeducation, cognitive restructuring, stress management and coping strategies, which constitute core components of the FASTER program. The observed improvements in self-worth may additionally reflect processes related to identity development and self-acceptance. A more positive autistic identity and greater self-acceptance have been associated with improved mental health outcomes, including lower depressive symptoms, whereas internalized stigma is linked to poorer self-concept and increased psychological distress (63–65). The group-based format may have facilitated shared experiences, mutual understanding, and normalization, thereby reducing self-stigma and supporting a more coherent and positive self-concept. Reductions in depressive symptoms may further be attributable to enhanced stress management and coping strategies, which are particularly relevant given the frequent co-occurrence of stress-related difficulties and depressive symptoms in autistic adults.

Taken together, these findings may suggest that structured, group-based psychosocial interventions can target proximal psychological outcomes, while broader improvements in overall quality of life may require longer-term or multimodal approaches that extend beyond individual-level treatment to support sustainable gains in daily functioning and well-being.

### 4.3 Summary

Overall, these findings align with the existing literature indicating that psychosocial interventions for autistic adults primarily impact proximal outcomes, such as social functioning, self-related constructs, and depressive symptoms, whereas more global aspects of well-being may require more sustained or multifaceted intervention approaches. Beyond acquiring social and stress-related knowledge and skills, the group setting may provide a unique opportunity for participants to share experiences with other autistic adults facing similar challenges, which may foster self-acceptance, support identity development, and reduce feelings of social isolation. Experiencing that modified behavioral strategies can positively influence social situations and coping with potentially stressful stimuli may further strengthen self-efficacy and encourage the application of these strategies in everyday life. From a clinical perspective, these processes may reflect the interplay between improved social competence, more positive social experiences, enhanced self-concept, and the impact of stress management strategies embedded in the intervention.

These findings support existing evidence, indicating that structured, group-based psychosocial interventions can enhance social functioning and self-related and emotional outcomes, thereby suggesting their potential clinical utility in routine care. Finally, the structured and manualized nature of the FASTER program may facilitate its implementation in routine clinical practice and contribute to the development of accessible psychosocial interventions for autistic adults.

## 5 Limitations and future directions

While this study provides important preliminary evidence for the implementation feasibility and clinical effectiveness of the FASTER group psychotherapy program under naturalistic conditions, several limitations should be considered.

First, the naturalistic design without a control group inherently limits causal inferences regarding the observed improvements. Given the uncontrolled pre–post design, the observed improvements should be interpreted with caution, as they cannot be unequivocally attributed to the intervention. A range of alternative explanations may account for the observed changes. For instance, expectancy effects and participants’ beliefs about treatment efficacy may have contributed to symptom improvement. In addition, regression to the mean may partially explain reductions in symptom severity, particularly among individuals with initially elevated scores. Natural fluctuations in symptomatology over time, independent of the intervention, also cannot be ruled out. Furthermore, non-specific therapeutic factors, such as therapist attention, group support, and common factors inherent to psychotherapeutic settings, may have played a role in the observed outcomes. As formal intervention fidelity assessments were not systematically conducted, conclusions regarding treatment adherence and consistency of implementation remain limited. Future controlled studies would benefit from more comprehensive fidelity monitoring procedures. While the high level of psychiatric comorbidity in the sample reflects clinical reality, it may also have introduced additional heterogeneity, potentially influencing treatment response. At the same time, the naturalistic clinical design enhances ecological validity by reflecting routine clinical practice.

Second, the relatively high dropout rate and the relatively low threshold for treatment exposure may have resulted in substantial heterogeneity in intervention exposure and a sample potentially biased toward individuals better able to engage with the intervention or manage daily life demands. Although the ≥25% attendance threshold was selected pragmatically to retain sufficient sample size under naturalistic routine care conditions, participants with lower attendance may have had fewer opportunities to benefit from the intervention, potentially attenuating observed intervention-related effects and limiting the interpretability of findings. In addition, participants who sustained treatment participation throughout the intervention may have differed from those with lower attendance in characteristics relevant to treatment engagement and outcomes, which should be considered when interpreting findings and their generalizability. Importantly, sensitivity analyses based on non-imputed observed data yielded highly comparable findings, supporting the robustness of the observed pattern of results despite missing data and attrition. At the same time, the finding that approximately 32% of participants attended fewer than 25% of sessions highlights challenges related to sustained participation and treatment retention, while underscoring the particular complexities of implementing group-based interventions for autistic adults under routine clinical care conditions, especially regarding social and emotional demands. Regarding implementation feasibility, the present study primarily evaluated implementation using pragmatic indicators such as treatment attendance, sustained participation, completion rates, and dropout. More comprehensive assessments of treatment acceptability, participant satisfaction, and intervention fidelity should be incorporated in future studies to enable a broader evaluation of feasibility. In addition, contextual factors related to everyday stress and overload may also have contributed to treatment discontinuation. Nevertheless, the naturalistic clinical design enhances ecological validity by reflecting routine clinical practice. Future interventions may benefit in particular from more intensive autism-specific preparation prior to group participation, including structured support for integrating the program into daily routines, in order to enhance adherence, reduce dropout rates, and minimize participant stress.

Third, regarding measurement, reliance on self-reported questionnaires may be limited in some participants due to the higher prevalence of alexithymia in autistic adults. Nevertheless, self-reported outcomes remain of particular clinical relevance for assessing perceived social functioning, well-being, and self-worth. While informant-based measures such as the SRS provide valuable information on observable social behavior, it also has some limitations. Informant ratings may be influenced by subjective perceptions, expectations, or the nature of the relationship with the participant, which could introduce bias. In addition, informants may not have access to all relevant social interactions, particularly those occurring outside their immediate observation. While informant reports are valuable for capturing observable aspects of social functioning, they may not fully reflect the individual’s subjective experience of social interactions. Accordingly, the absence of a corresponding self-report measure limits the ability to examine potential discrepancies between internal and external perspectives on social functioning. Although originally developed for children and adolescents, the SRS has since been validated and applied across the lifespan, including in autistic adult populations. Obtaining informant reports from relatives, partners, or friends was not feasible for some participants, reflecting the common social circumstances of autistic adults, such as living alone or having limited social networks. Consequently, the return rate for informant-report questionnaires was limited. In cases where no informant report was available, participants self-reports were collected but excluded from analyses, given that the SRS was designed primarily as an informant-report measure. Specifically, the ancillary, adapted version of the SRS (SRS mod) used to assess perceived changes in social reactivity from baseline to post-intervention has not yet undergone formal psychometric validation, which inherently constrains its interpretability and comparability. Consequently, rather than providing confirmatory evidence of efficacy, findings derived from this exploratory measure must be interpreted with caution and regarded as strictly hypothesis-generating. Nonetheless, it seems to capture subjective changes more precisely than the original SRS in a pre–post intervention assessment.

Fourth, the sample consisted exclusively of autistic adults without intellectual disability treated in a single-center, specialized outpatient setting. This may limit the generalizability of the findings to other populations or clinical contexts. Future research should explore which subgroups of autistic adults - particularly regarding age, baseline social functioning, or sex - benefit most from intervention, and which specific intervention components are most effective. Given the relatively limited number of female participants, the present study was not sufficiently powered to examine potential sex-specific differences in treatment response. Future studies with larger and more balanced samples are needed to investigate potential sex-specific treatment needs and outcomes more systematically. In addition, age at autism diagnosis was not assessed in the present study and should be considered in future research, as it may influence subsequent psychopathology, identity development, compensatory strategies, and vulnerability to personality-related difficulties. Personality disorders were not systematically assessed in this study and should likewise be considered in future research, given their potential influence on treatment response.

Finally, additional research is needed to clarify the mechanisms underlying treatment-related changes, identify potential moderators of response, and determine the active components driving observed effects. Multi-center trials, longer follow-up periods, and randomized controlled designs are particularly necessary to replicate and extend these findings, evaluate the stability of treatment effects over time, and increase generalizability.

To conclude, while this study provides promising preliminary evidence for the implementation feasibility and effectiveness of the FASTER program, rigorous controlled trials are required to confirm these results and inform implementation in broader clinical settings.

## 6 Summary and conclusions

In summary, this open, naturalistic, clinical study provides preliminary evidence for the implementation feasibility and clinical effectiveness of the FASTER group psychotherapy program for autistic adults without intellectual disability. The findings indicate that the intervention is feasible in a routine clinical care setting and are associated with improvements in social responsiveness, self-worth, and depressive symptoms.

Despite the methodological limitations inherent to naturalistic study designs, the results highlight the potential of structured, manualized group interventions to address core social and emotional challenges in autistic adults. Given the limited availability of evidence-based psychosocial interventions for this population, the FASTER program represents a promising and clinically relevant approach.

Further controlled, randomized, multi-center studies with larger samples are needed to confirm these findings and to establish the program as an evidence-based treatment option.

## Data Availability

The raw data supporting the conclusions of this article will be made available by the authors, without undue reservation.
